# Secreted Isoform of Human Lynx1 (SLURP-2): Spatial Structure and Pharmacology of Interactions with Different Types of Acetylcholine Receptors

**DOI:** 10.1038/srep30698

**Published:** 2016-08-03

**Authors:** E. N. Lyukmanova, M. A. Shulepko, Z. O. Shenkarev, M. L. Bychkov, A. S. Paramonov, A. O. Chugunov, D. S. Kulbatskii, M. Arvaniti, Eva Dolejsi, T. Schaer, A. S. Arseniev, R. G. Efremov, M. S. Thomsen, V. Dolezal, D. Bertrand, D. A. Dolgikh, M. P. Kirpichnikov

**Affiliations:** 1 Lomonosov Moscow State University, Leninskie Gori 1, Moscow 119234, Russian Federation; 2 Shemyakin-Ovchinnikov Institute of Bioorganic Chemistry RAS, Miklukho-Maklaya Street 16/10, Moscow 117997, Russian Federation; 3 Moscow Institute of Physics and Technology, Institutskiy Pereulok 9, Dolgoprudny, Moscow Region 141700, Russian Federation; 4 Department of Drug Design and Pharmacology, University of Copenhagen, Jagtvej 160, DK-2100 Copenhagen, Denmark; 5 Institute of Physiology, Academy of Sciences of the Czech Republic (public research institution), Prague, 14220, Czech Republic; 6 HiQScreen Sàrl, 6 rte de Compois, 1222, Vésenaz, Geneva, Switzerland; 7 National Research University Higher School of Economics, Myasnitskaya ul. 20, 101000 Moscow, Russia

## Abstract

Human-secreted Ly-6/uPAR-related protein-2 (SLURP-2) regulates the growth and differentiation of epithelial cells. Previously, the auto/paracrine activity of SLURP-2 was considered to be mediated via its interaction with the α3β2 subtype of the nicotinic acetylcholine receptors (nAChRs). Here, we describe the structure and pharmacology of a recombinant analogue of SLURP-2. Nuclear magnetic resonance spectroscopy revealed a ‘three-finger’ fold of SLURP-2 with a conserved β-structural core and three protruding loops. Affinity purification using cortical extracts revealed that SLURP-2 could interact with the α3, α4, α5, α7, β2, and β4 nAChR subunits, revealing its broader pharmacological profile. SLURP-2 inhibits acetylcholine-evoked currents at α4β2 and α3β2-nAChRs (IC_50_ ~0.17 and >3 μM, respectively) expressed in *Xenopus* oocytes. In contrast, at α7-nAChRs, SLURP-2 significantly enhances acetylcholine-evoked currents at concentrations <1 μM but induces inhibition at higher concentrations. SLURP-2 allosterically interacts with human M1 and M3 muscarinic acetylcholine receptors (mAChRs) that are overexpressed in CHO cells. SLURP-2 was found to promote the proliferation of human oral keratinocytes via interactions with α3β2-nAChRs, while it inhibited cell growth via α7-nAChRs. SLURP-2/mAChRs interactions are also probably involved in the control of keratinocyte growth. Computer modeling revealed possible SLURP-2 binding to the ‘classical’ orthosteric agonist/antagonist binding sites at α7 and α3β2-nAChRs.

The Ly-6/uPAR family includes proteins with a characteristic ‘three-finger’ fold composed of a compact β-structural core (‘head’) and three protruding loops (‘fingers’) that are stabilized by a system of invariant disulfide bonds^1^ (Fig. 1A,E). The prototypical Ly-6/uPAR members, snake ‘three-finger’ toxins, act on numerous targets, including GABA_A_-receptors^2^,^3^, nicotinic acetylcholine receptors^4^,^5^ (nAChRs), and muscarinic acetylcholine receptors^6^,^7^ (mAChRs). Both types of acetylcholine receptors, which belong to the different families of membrane proteins (ligand-gated ion channels and G-protein coupled receptors, GPCRs), are responsible for cholinergic signaling not only in the central and peripheral nervous systems but also in non-neuronal tissues including epithelial and immune cells^8^.

Many endogenous Ly-6/uPAR proteins have been discovered in the nervous, endocrine, and immune systems of higher animals^9^. Some Ly-6/uPAR proteins (for example, Lynx1) are membrane-tethered via GPI anchors and co-localize with nAChRs, thus modulating the function of receptors in the brain^10^. At present, Lynx1 is considered an important factor that regulates neuronal plasticity^11^,^12^. In contrast, secreted Ly-6/uPAR-related proteins (SLURP-1 and SLURP-2) are expressed by epithelial and immune cells^13^,^14^,^15^, and probably also by sensory neurons^16^. At present, SLURPs are considered autocrine/paracrine regulators that control the growth, differentiation, inflammation, and malignant transformation of epithelial cells^17^,^18^. SLURPs influence the healing process of dermal and mucosal wounds^19^, and they demonstrate anti-inflammatory effects on human intestinal epithelial cells and immunocytes^20^. In addition, SLURP-2 expression in human keratinocytes is regulated by cytokines^21^. In accordance with their proposed role, SLURPs participate in the development of several pathologies. Point mutations in the human *slurp-1* gene or knock-out of the *slurp-2* gene causes the autosomal inflammatory skin disease Mal de Meleda^22^,^23^. Gene expression of *slurp-2* is up-regulated threefold in psoriatic lesional skin compared with the skin of healthy individuals^14^. Notably, SLURP-2 represents a secreted isoform of the membrane-tethered neuromodulator Lynx1 mentioned above. Both SLURP-2 and Lynx1 are produced by the *lynx1* gene (OMIM: 606110) by alternative splicing. SLURP-2 and Lynx1, however, demonstrate a small degree of sequence homology (~32%, Fig. 1A). The homology between SLURP-2 and SLURP-1 is also not very high (~29%, Fig. 1A). Notably, the ten invariant Cys residues are responsible for 10–15% of the sequence identity between different Ly-6/uPAR proteins.

In studies investigating the immortalized line of human oral keratinocytes (cell line Het-1A), SLURP-1 has been shown to inhibit cell proliferation via interactions with α7 nAChRs^13^,^24^, while SLURP-2 promotes cell growth. This effect is likely mediated by non-α7 (e.g., α3β2) receptor subtypes^25^. Notably, oral keratinocytes express several types of nAChR subunits (α3, α5, α7, α9, α10, β2, β4) and M2-M5 types of mAChRs^26^. Cholinergic receptors regulate cell adhesion, migration, cell cycle progression, apoptosis and differentiation, and their repertoire evolves during cell development^26^.

Recently, using human colorectal adenocarcinoma HT-29 cells, it was shown that rSLURP-2 as well as rSLURP-1 can inhibit the proliferation of epithelial cells^27^. HT-29 cells contain mRNAs that encode only the α4, α5, α7 and β1 nAChR subunits, and only α7 subunits of this set can form functional receptors^28^. It is likely that SLURP-2 has multiple molecular targets and that the inhibitory effect of rSLURP-2 in HT-29 cells results from its interaction with α7 nAChRs^27^. Although nAChRs have been suggested to be targets of SLURP-2, there is currently no direct evidence of SLURP-2/nAChR interactions.

Here, we describe, for the first time, the functional and structural properties of a recombinant analogue of human SLURP-2 (rSLURP-2), which differs from the native protein by only one additional residue (*N*-terminal Met, which appears due to translation of the starting *atg* codon). We show that rSLURP-2 acts on a broad range of nAChRs subtypes, not only on the α3β2 receptor, and we describe a new target of SLURP-2, mAChRs. The overall effect of rSLURP-2 on keratinocyte proliferation likely represents a combination of the contributions from both types of cholinergic signaling. The high-resolution spatial structure and backbone dynamics of rSLURP-2 in solution were determined by nuclear magnetic resonance (NMR) spectroscopy, which allowed us to model SLURP-2 complexes with ligand-binding domains of α7 and α3β2 nAChRs that provided structural insights into interactions of SLURP-2 with some of its targets. The obtained results revealed some similarities and unique structure-functional properties of SLURP-2 compared with other Ly-6/uPAR proteins, including SLURP-1 and Lynx1.

## Results

### Spatial structure, backbone dynamics and aggregation state of rSLURP-2 in solution

**Figure 1:**
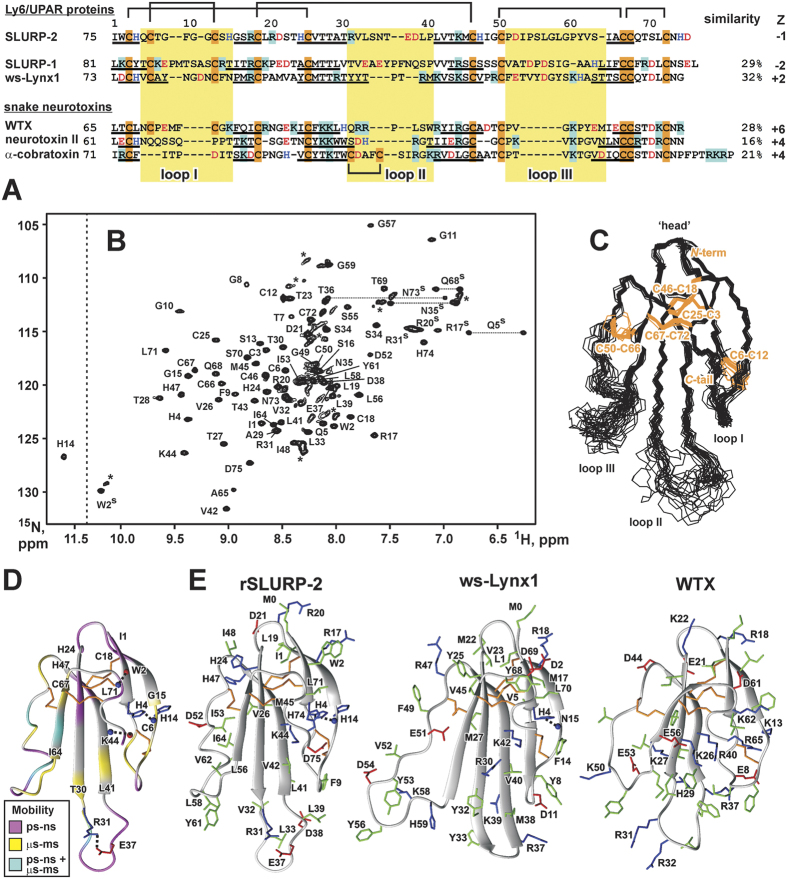
Amino acid sequence alignment and comparison of the spatial structure of SLURP-2 with other human Ly-6/uPAR proteins and three-finger snake neurotoxins. (**A**) The sequence of the water-soluble domain of human Lynx-1 (ws-Lynx1) is shown without the GPI consensus sequence at the C-terminus. The positively charged (Arg/Lys), negatively charged (Asp/Glu), and His and Cys residues are highlighted. The fragments corresponding to β-strands in the spatial structures of the proteins are underlined. The loop regions are highlighted with a yellow background. (**B**) The 2D ^1^H,^15^N-HSQC spectrum of rSLURP-2 (0.5 mM, 5% dioxane, pH 5.0, 37 °C). The obtained resonance assignments are shown. The resonances of the side-chain groups are indicated by a superscripted “s”. The signals corresponding to the unfolded/aggregated protein are marked by asterisks. The relative population of this protein form did not exceed 10%. (**C**) The set of the best 20 rSLURP-2 structures were superimposed over the backbone atoms in regions with a well-defined structure. The three loops and ‘head’ of the protein are labeled. Disulfide bonds are shown in orange. (**D**) Ribbon representation of the spatial structure of rSLURP-2 with mapped dynamic NMR data. ^15^N relaxation rates were measured at 60 MHz (pH 5.0, 37 °C) for 0.08 mM rSLURP-2 in water without the addition of dioxane (Supplementary Fig. S4). The backbone fragments affected by dynamic processes on the ps-ns time scale (heteronuclear NOE < 0.7) or μs-ms time scale (R_1_ · R_2_ product > 20 s^−2^ or HN signals broadened beyond the detection limit) are highlighted. Additional electrostatic and hydrogen bonding interactions that stabilize the protein fold are shown. Backbone amide and carbonyl groups are indicated by blue and red spheres, respectively. (**E**) Comparison of the spatial structures of rSLURP-2, ws-Lynx1 and the WTX[P33A] mutant. Aromatic/hydrophobic, positively charged (including His), negatively charged, and Cys residues are indicated in green, blue, red, and orange, respectively.

The engineering of a bacterial expression system (see ref. 27 and Supplementary Fig. S1) allowed us to obtain a ^13^C-^15^N-labeled sample of rSLURP-2 and to study its spatial structure by heteronuclear NMR spectroscopy. Initial screening of the optimal conditions for the NMR study revealed that rSLURP-2 (calculated pI value of ~6.5) at sub-millimolar concentrations is stable in solution only within a narrow pH range (4.5–6.0) (see Supplementary text and Fig. S2). Lowering the pH below 4.5 resulted in reversible unfolding of the β-structural core of the protein. Increasing the pH above 6.0 (0.1 mM sample) or increasing the rSLURP-2 concentration above 0.2 mM at pH ~5.0 led to protein precipitation or reversible aggregation, respectively. To reduce protein oligomerization at pH ~5.0, 5% dioxane was added to the sample, which greatly improved the quality of the NMR spectra and permitted studies of the protein spatial structure in solution using the 0.5 mM rSLURP-2 sample (Fig. 1B). Comparison of the obtained ^1^H and ^15^N chemical shifts with those of the 0.08 mM protein in water revealed that the addition of dioxane did not perturb the spatial structure of rSLURP-2 (Supplementary Fig. S3).

Our NMR data revealed that rSLURP-2 adopts a typical ‘three-finger’ fold consisting of two antiparallel β-sheets (Fig. 1C,E). The first β-sheet is formed by two β-strands and involves residues from loop I (Ile1-His4, Gly15-Cys18). The second sheet consists of three strands that are formed by the residues from loop II (His24-Thr30, Leu41-His47) and loop III (Ile64-Cys67). Similar to the other ‘three-finger’ proteins^29^, rSLURP-2 encompasses several conserved β-turns in the ‘head’ region and in the *C*-terminal ‘tail’ (‘*C*-tail’, Supplementary Fig. S3). The rSLURP-2 molecule is stabilized by four disulfide bonds in the “head” (Cys3-Cys25, Cys18-Cys46, Cys50-Cys66 and Cys67-Cys72), and one in the first loop (Cys6-Cys12). A similar disulfide arrangement has been observed in other mammalian Ly-6/uPAR proteins and in ‘non-conventional’ snake neurotoxins, e.g., in a ‘weak’ toxin from *Naja kaouthia* (WTX) (Fig. 1A,E). Apart from the backbone-backbone hydrogen bonds associated with canonical elements of the secondary structure, the “head” and loops of the protein are stabilized by additional H-bonding and electrostatic interactions (Fig. 1D). For example, two hydrogen bonds (HN Leu71–CO Trp2 and HN Lys44–CO Cys6) control the spatial arrangement of loop I relative to ‘*C*-tail’ and loop II of the protein, respectively. The potential salt bridge (Arg31–Glu37) and hydrogen bond (HN His14 - N^δ1^ His4) cross-link loops II and I, respectively. Interestingly, the similar N-H∙∙∙N hydrogen bond has been previously observed in the structure of the water-soluble domain of human Lynx-1 (ws-Lynx, Fig. 1E). Formation of this characteristic side-chain/main-chain bond leads to a significant downfield shift of the corresponding ^1^H^N^ resonance (~11.6 ppm, Fig. 1B).

The spatial structure of SLURP-2 is well defined only in the conserved β-structural core, while the tips of all three loops of the protein are disordered (Fig. 1C). The obtained ^15^N relaxation data (Fig. 1D and Supplementary Fig. S4) revealed that the absence of the structural convergence in these regions is a consequence of the enhanced mobility of the protein backbone on the two time scales (picosecond to nanosecond and microsecond to millisecond). Notably, to minimize the influence of protein aggregation and dioxane addition on the rates of ^15^N relaxation, the measurements were performed for the 0.08 mM protein sample in water without dioxane. Analysis of the obtained relaxation data also confirmed that rSLURP-2 is monomeric in water at concentrations below 0.1 mM (Supplementary Fig. S4).

### rSLURP-2 can bind to multiple nAChR subtypes in brain extracts

**Figure 2:**
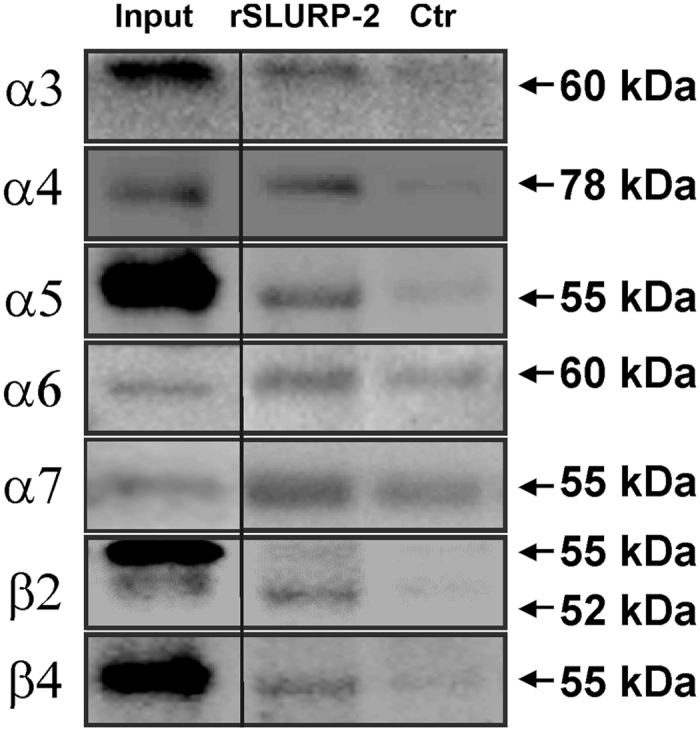
rSLURP-2 binds to different nAChR subunits in human brain extracts. Affinity purification was performed with rSLURP-2 that was covalently coupled to magnetic beads or non-coupled beads (Ctrl) using human temporal cortical homogenates. The samples were subjected to gel electrophoresis and Western blotting along with the homogenate samples used for affinity purification (Input), followed by the detection of nAChR subunits. Representative blot images obtained in one (of two) independent experiment are shown. The representative blot images from the second experiment are shown in Supplementary Fig. S5. The two bands were observed for the β2 subunit. As shown previously, only the lower band corresponded to the β2 subunit^55^.

To confirm that nAChRs are the target of SLURP-2 and to initially characterize the possible pharmacological spectrum of rSLURP-2 activity, we chose a model system with a large density of receptors, namely brain extracts. Affinity purification of nAChR subunits from human temporal cortex extracts was performed using bead-coupled rSLURP-2 followed by Western blot analysis. The specific interaction of nAChR subunits with rSLURP-2 was confirmed in experiments with control beads without coupled rSLURP-2 (Fig. 2 and Supplementary Fig. S5). Despite the presence of some nonspecific binding observed for non-coupled beads, we concluded that rSLURP-2 could associate with the α3, α4, α5, α6, α7, β2, and β4 subunits (Fig. 2 and Supplementary Fig. S5). For the α6 subunit, we identified only a small difference in the band intensities between rSLURP-2-coupled beads and control beads (n = 2); thus, we cannot determine the specificity of the binding to this subunit. Notably, previously conducted experiments with the other secreted Ly-6/uPAR protein (rSLURP-1) revealed specific extraction only of the α7 nAChR subunit from the brain extracts^24^.

### Electrophysiology studies of rSLURP-2 interactions with human nAChRs

**Figure 3:**
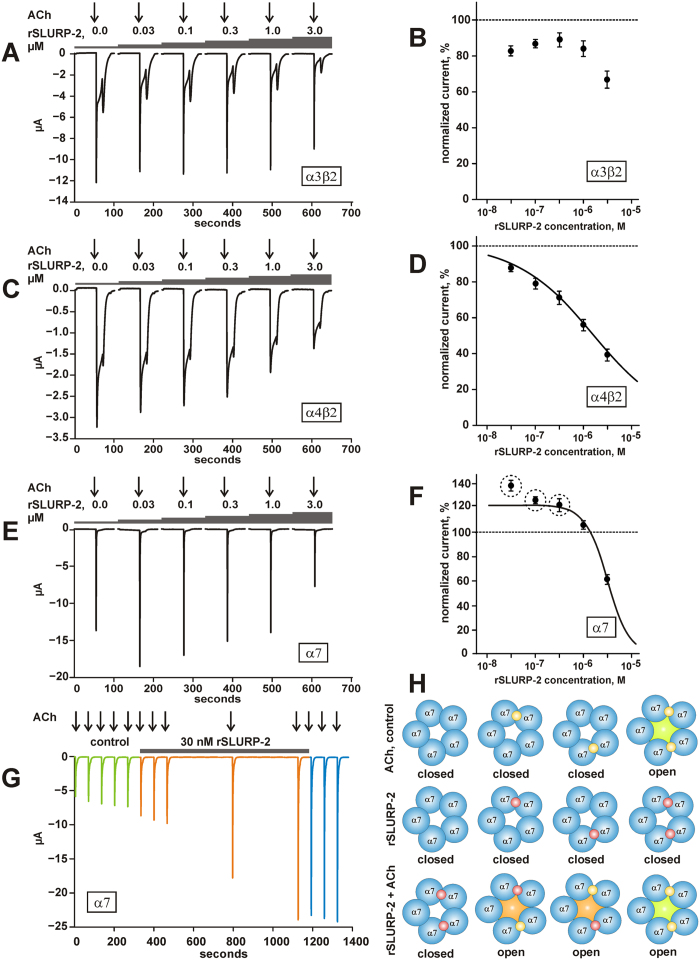
Effect of rSLURP-2 on nAChRs expressed in *Xenopus* oocytes. (**A,C,E**) Electrophysiological recordings of currents evoked by 100, 10, and 100 μM of ACh at α3β2, α4β2, and α7 nAChRs, respectively. The concentrations of ACh were similar to the half activation concentrations (EC_50_) of the corresponding receptor subtypes^56^. The 5-second pulses of ACh are indicated by the arrows above the traces. The concentration of rSLURP-2 is indicated above the traces. (**B,D,F**) Dose-response curves of ACh-evoked currents by rSLURP-2 at the α3β2, α4β2, and α7 nAChRs. Each point represents the average ± S.E. of seven, six and seven independent experiments, respectively. The Hill equation (y = A0/(1+([rSLURP-2]/IC_50_)^nH^)) was fitted to the normalized data (% of control) obtained at the α4β2 and α7 receptors. For the α4β2 receptor, the value of the scaling parameter (A0) was fixed at 100%. The calculated A0 value for the α7 receptor was 122 ± 3%. The calculated IC_50_ and nH parameters were 1.7 ± 0.4 μM and 0.54 ± 0.03, and 3.0 ± 0.2 μM and 1.90 ± 0.14, for the α4β2 and α7 receptors, respectively. (**G**) Electrophysiological recordings of the ACh-evoked current at α7 nAChR in the absence and presence of 30 nM rSLURP-2. Currents were elicited by 5-second pulses of 40 μM ACh. ACh pulses were interrupted by periods of silence. A representative trace from nine independent experiments is shown. Green traces represent the responses evoked by ACh in the absence of the compound, orange traces are the responses evoked by the same ACh test pulse in the presence of rSLURP-2, and blue traces are the responses evoked by ACh after terminating the rSLURP-2 application. (**H**) Cartoon demonstrating the priming activity of SLURP-2 at α7 nAChRs. ACh and SLURP-2 molecules are indicated as yellow and red spheres, respectively. The open α7 nAChR channels are indicated by green and orange cores. Closed channels have a white core.

To confirm the interactions of rSLURP-2 with nAChRs and to characterize rSLURP-2 effects on several widely distributed and important receptor subtypes, electrophysiological recordings were performed using *Xenopus* oocytes expressing human α4β2, α3β2, and α7 nAChRs. The amplitude of ACh-evoked currents recorded in the absence of rSLURP-2 was compared with the currents observed in the presence of 30 nM–3 μM of the protein. The recorded traces (Fig. 3A,C,E) revealed that the application of rSLURP-2 alone did not elicit currents at any of the tested receptors. Exposure to rSLURP-2 caused a moderate inhibitory effect on ACh-evoked currents at α3β2 nAChR (IC_50_ > 3 μM), but a clear concentration-dependent inhibition of the α4β2 receptor was observed (IC_50_ ~1.7 μM) (Fig. 3A–D, respectively). Similarly, rSLURP-2 inhibited the α7 receptor with an apparent IC_50_ of ~3 μM. At low concentrations (<1 μM), however, the response evoked by ACh at the α7 receptor was enhanced (priming effect) by the presence of rSLURP-2.

To further evaluate a possible priming effect caused by rSLURP-2, experiments with irregular stimulation of the receptors were designed. As shown in Fig. 3G (n = 9), exposure to 30 nM rSLURP-2 resulted in a potentiation of the ACh-evoked current through α7 nAChRs. Potentiation of the currents observed using this experimental protocol corresponded to an enhanced ACh response in the dose-response curve (Fig. 3F), however, amplitude of the effect was significantly larger.

### rSLURP-2 affects nicotine-induced ERK1/2 MAP kinase phosphorylation

**Figure 4:**
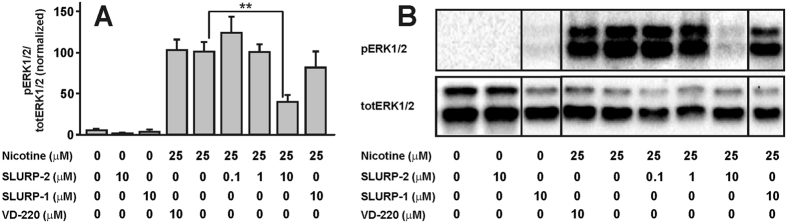
SLURP-2 reduces nicotine-mediated phosphorylation of ERK1/2 in PC12 cells. (**A**) An increase in ERK1/2 phosphorylation in PC12 cells was elicited by 25 μM nicotine, and this effect was blocked by pre-incubation with 10 μM recombinant SLURP-2. Values are presented as the ratio of phosphorylated (pERK1/2) to total ERK1/2 (totERK1/2) protein and are normalized to the 25 μM nicotine group (n = 8, mean ± S.E). **indicates a significant difference (p < 0.01, ANOVA followed by Dunnett’s multiple comparisons test) compared with the 25 μM nicotine group. (**B**) Representative images of Western blots summarized in (**A**).

PC12 cells were used to further study the spectrum of rSLURP-2 activity and to investigate its possible intracellular effects. This cell line along with expression of adenosine, dopamine, and muscarinic receptors also expresses α3, α5, α7, β2 and β4 subunits of nAChRs. PC12 cells are frequently used as model neuron-like cells to study the influence of different ligands on nAChR-mediated intracellular signaling^30^,^31^. For example, exposure of PC12 cells to nicotine induces the phosphorylation of ERK1/2 MAP kinases, and this effect is mediated by activation of α3β4 nAChRs^30^. Pre-incubation of PC12 cells with 10 μM rSLURP-2 significantly inhibited ERK1/2 phosphorylation induced by exposure to 25 μM nicotine (up to 60%, Fig. 4A,B). The application of lower rSLURP-2 concentrations (0.1 and 1 μM) did not significantly diminish nicotine-induced MAP kinase phosphorylation (Fig. 4), likely indicating a very low (10 μM range) rSLURP-2 affinity for the α3β4 receptor. The specificity of the effects of rSLURP-2 on α3β4 nAChR-mediated MAP kinase phosphorylation was confirmed in control experiments with two peptides that do not act on this receptor subtype. As expected, 10 μM of VD-220 peptide (RYHHHDPDGG) or 10 μM rSLURP-1 did not significantly influence nicotine-induced ERK1/2 phosphorylation. Notably, rSLURP-2 (as well as rSLURP-1) exposure alone did not affect the basal levels of ERK1/2 phosphorylation (Fig. 4A,B).

### rSLURP-2 affects keratinocyte growth via interactions with different types of acetylcholine receptors

**Figure 5:**
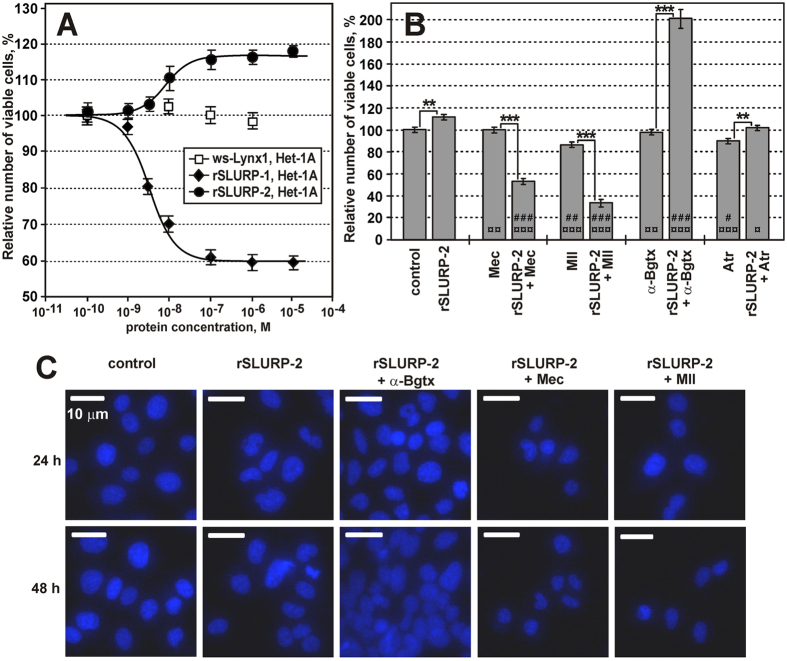
Effects of rSLURP-2 on the growth of Het-1A cells. (**A**) Influence of rSLURP-2, rSLURP-1, and ws-Lynx1 on Het-1A cell growth (% of control, n = 6–12, mean ± S.E.). The Hill equation (y = A1(100%-A1)/(1+([protein]/IC_50_)^nH^)) was fitted to the data measured using WST-1 reagent. The data for rSLURP-1 were obtained from ref. 24. The calculated EC_50_, nH and A1 parameters were 7.6 ± 1.0 nM, 1.5 ± 0.4 and 116 ± 1% (rSLURP-2/Het-1A), and 4.3 ± 0.6 nM, 1.4 ± 0.2 and 60 ± 1% (rSLURP-1/Het-1A), respectively. (**B**) Effects of rSLURP-2 (1 μM), Atr (1 μM), Mec (10 μM), α-Bgtx (1 μM), MII (1 μM) and their co-application on the growth of Het-1A cells after 48 hours. Each bar is the mean ± S.E. of four independent experiments performed in triplicate. The pairwise statistical analysis of the data groups measured with and without rSLURP-2 was done using t-test. Data indicated as **(p < 0.01) and ***(p < 0.001) are significantly different from each other. Multiple comparisons of all the data groups with the ‘control’ group and with the ‘rSLURP-2’ group were done using ANOVA followed by special Dunnett´s multiple comparisons test. Data marked with ^¤^ and ^#^(p < 0.05), ^¤¤^ and ^##^(p < 0.01), ^¤¤¤^ and ^###^(p < 0.001) are significantly different from ‘rSLURP-2’ and the ‘control’, respectively. (**C**) Effects of rSLURP-2 (1 μM) and its co-application with atropine (Atr, 1 μM), Mec (10 μM), α-Bgtx (1 μM), or α-conotoxin MII (MII, 1 μM) on the morphology of Het-1A cell nuclei after 24 and 48 hours. The cells nuclei were colored with Hoechst 33342 and propidium iodide.

Incubation of Het-1A cells (an immortalized human oral keratinocyte cell line) with ≥10 nM rSLURP-2 led to a slight, but significant, increase in the number of cells up to ~116% relative to the control (p < 0.01, n = 12, Fig. 5A). The dose-response curve analysis revealed the concentration-dependent mode of rSLURP-2 activity with an EC_50_ of ~8 nM (Fig. 5A). To study the role of different nAChR subtypes in the regulation of keratinocyte proliferation via SLURP-2, we used selective inhibitors of α7 and α3β2 receptors, α-Bgtx and α-conotoxin MII, respectively, and the non-specific inhibitor of the nAChR, mecamylamine (Mec). α-Bgtx and Mec did not significantly influence cell growth per se, but α-conotoxin MII decreased the number of viable cells to ~87% relative to the control (Fig. 5B). Pre-incubation of keratinocytes with α-Bgtx resulted in an approximately two-fold increase in the number of viable cells in the presence of rSLURP-2 (Fig. 5B). In contrast, pre-incubation with α-conotoxin MII and Mec resulted in a pronounced decrease in the number of viable cells in the presence of rSLURP-2 (down to 34 ± 4% and 54 ± 3% relative to the control, respectively) (Fig. 5B). To discriminate between cytotoxicity and reduced proliferation, we performed an additional microscopic examination of cells and measured their viability using the Hoechst/propidium iodide assay (see ‘Methods’). A decrease in cell density was clearly observed, while the morphology of most cells and their nuclei were not modified compared with the control (representative images are shown in Fig. 5C, and calculated cell numbers are presented in Supplementary Fig. S6). The Hoechst/propidium iodide assay did not reveal an increase in the fraction of dead cells (4 ± 1% of dead cells for both treated and control cells). Therefore, co-application of MII and Mec with rSLURP-2 switched its activity from proliferative to antiproliferative.

The marked antiproliferative effect observed upon simultaneous application of Mec (non-specific nAChR inhibitor) and rSLURP-2 suggested the presence of other SLURP-2 targets in keratinocytes. To examine a possible interaction of rSLURP-2 with mAChRs, which are expressed in keratinocytes along with nAChRs^26^, we studied rSLURP-2 activity in the presence of atropine, a competitive non-specific mAChR antagonist (Fig. 5B). Exposure of cells to 1 μM atropine led to a small, but significant, reduction in the number of viable keratinocytes to ~91% relative to the control (Fig. 5B). Upon the simultaneous application of atropine with rSLURP-2, the antiproliferative and proliferative effects of these compounds neutralized one another (number of viable cells ~102%, which is significantly different from both ‘rSLURP-2’ and ‘atropine’, p < 0.05, n = 4, Fig. 5B). The apparent additivity of the effects of rSLURP-2 and atropine did not permit an unequivocal conclusion regarding the influence of atropine on rSLURP-2 activity.

### rSLURP-2 allosterically interacts with M1 and M3 mAChRs

**Figure 6:**
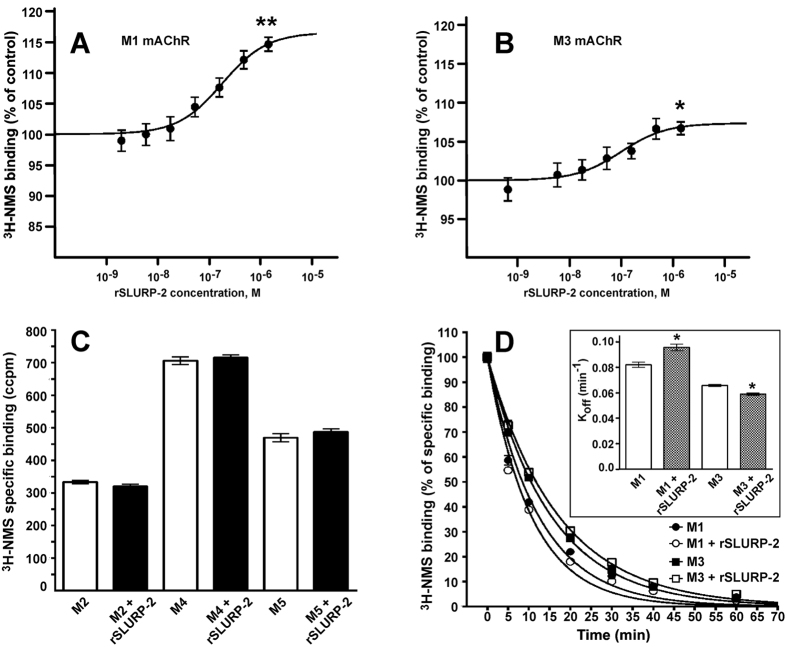
Influence of rSLURP-2 on [^3^H]-NMS binding to mAChRs. (**A,B**) Interaction of rSLURP-2 with ^3^H-NMS binding at M1 and M3 receptors, respectively. Membranes (20 μg of protein) were incubated in the presence of the indicated concentrations of rSLURP-2 and 100 pM ^3^H-NMS. ^3^H-NMS binding is expressed as the percent of control binding in the absence of rSLURP-2. Data points are means ± S.E. of four independent experiments performed in quadruplicate. The equation y = 100 * ([NMS]+K_d_)/{[NMS]+K_d_·(K_a_+[rSLURP-2])/(K_a_+[rSLURP-2]/α)} was fitted to normalized data. The K_d_ of ^3^H-NMS binding (197 and 187 pM for M1 and M3 mAChRs, respectively, see Supplementary Table S2) was determined in parallel saturation experiments. The calculated K_a_ (equilibrium dissociation constant of rSLURP-2) and α (factor of cooperativity) parameters were 231 ± 81 nM and 1.296 ± 0.051, and 144 ± 56 nM and 1.128 ± 0.013, for the M1 and M3 receptors, respectively. *and **indicate significant differences compared with the control (p < 0.05, p < 0.01, respectively, t-test). (**C**) Interaction of rSLURP-2 with ^3^H-NMS binding at M2, M4, and M5 receptors. Membranes expressing 5–20 μg of protein were incubated in the presence of 1.4 μM rSLURP-2 and 150 pM ^3^H-NMS. Bars represent the mean ± S.E. of specific ^3^H-NMS binding in two independent experiments performed in quadruplicate. (**D**) Influence of rSLURP-2 on the dissociation rate of [^3^H]-NMS at M1 and M3 mAChRs. Time course of [^3^H]-NMS dissociation in the presence and absence of rSLURP-2 was determined as described in Methods. Specific binding of [^3^H]-NMS was calculated as the difference between the total and non-specific binding measured in the same experiment and is expressed as the percent of initial binding (ordinate). The exponential decay equation was fitted to the normalized data (% of control binding). The (insert) 4.2 μM rSLURP-2 changed the [^3^H]-NMS dissociation rate constants (K_off_, min^−1^) from 0.082 ± 0.002 to 0.093 ± 0.002 (n = 4, p < 0.05, t-test) and from 0.066 ± 0.001 to 0.059 ± 0.002 (n = 4, p < 0.05, t-test) at the M1 and M3 receptors, respectively. The dissociation rate constants are presented as the means ± S.E. of the values obtained in four independent experiments performed in triplicate.

Direct investigation of the interaction of rSLURP-2 with mAChRs in keratinocytes using affinity purification and Western blot analysis was unsuccessful, probably due to a low receptor density in cultured keratinocytes^32^. Therefore, to study the ability of rSLURP-2 to interact specifically with the different human mAChR subtypes, we used the membranes of CHO cells overexpressing individual subtypes of the receptor. A weak positive influence of rSLURP-2 on ^3^H-N-methyl-scopolamine (NMS) binding was observed in a pseudocompetition experiment at M1 and M3 receptors (equilibrium dissociation constant of K_a_ ~230 and 140 nM, respectively, Fig. 6A,B). In contrast, no competition at M2, M4, and M5 receptors was observed with NMS at relatively large concentration of rSLURP-2 (1.4 μM) (Fig. 6C).

To discriminate between orthosteric and allosteric modes of the interaction of rSLURP-2 with mAChRs, its effect on the rate of [^3^H]-NMS dissociation was measured at M1 and M3 receptor subtypes (Fig. 6D). The dissociation rates of the orthosteric antagonist [^3^H]-NMS in the presence of rSLURP-2 were modified compared with the control experiments (n = 4, p < 0.05, Fig. 6D, insert). The dissociation rate (min^−1^) increased from 0.082 ± 0.002 to 0.093 ± 0.002 (n = 4, p < 0.05) at the M1 receptor, and decreased from 0.066 ± 0.001 to 0.059 ± 0.002 (n = 4, p < 0.05) at the M3 receptor (Fig. 6D). These data support an allosteric nature of the rSLURP-2/mAChR interactions.

### Computer modeling of the interaction of rSLURP-2 with α7 and α3β2 nAChRs

Our finding that the effects of rSLURP-2 are altered in the presence of selective orthosteric antagonists of nAChRs (Fig. 5B) suggests a probable overlap of the rSLURP-2 binding site on the receptor surface with the orthosteric binding site. The orthosteric binding site (‘classical’ binding site) is situated in the extracellular portion of the receptor at the interface of the principal (+) and complementary (−) subunits, and it involves loop C of the principal subunit^33^. Thus, we performed protein-protein docking of rSLURP-2 to the water-soluble extracellular domains of α7 and α3β2 nAChRs based on the assumption that, upon binding to nAChRs, rSLURP-2 interacts with loop C of the principal subunit.

To consider the structural flexibility of both partners, molecular dynamic (MD) simulations in an explicit water box were initially performed separately for rSLURP-2 and the receptor domains. Subsequent clustering of the resulting conformations yielded conformational ensembles, which were further used in the protein-protein docking calculations. For the α7 nAChRs, the conformation of loop C was substantially distinct for the different subunit interfaces in the obtained structural ensembles. Conformations with ‘closed’ and ‘open’ binding pockets differed by the position of the loop C of the principal subunit. In contrast, in α3β2 receptors, both binding pockets had a ‘closed’ configuration of loop C. Clustering of the α3β2 (‘closed’), α7 (‘closed’), and α7 (‘open’) subunit interfaces yielded 9, 9 and 11 structures, respectively. In each group of structures, the most substantial differences were observed in the region of loop C and the adjacent loops. Conformational clustering of rSLURP-2 yielded 11 representative structures.

**Figure 7:**
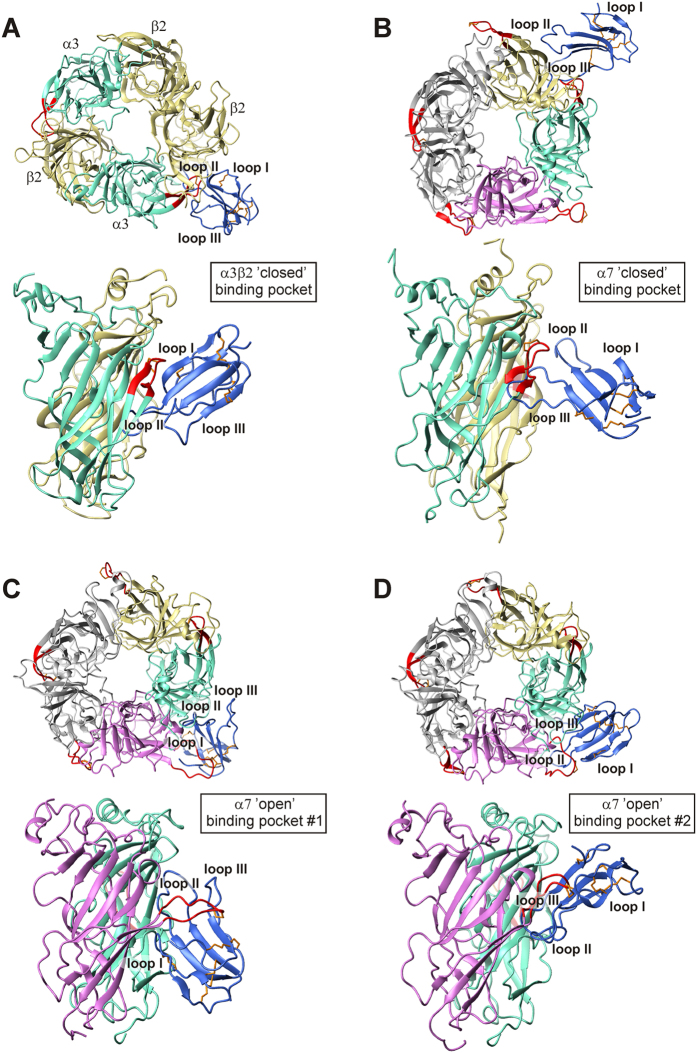
Modeled complexes of rSLURP-2 with α3β2 and α7 nAСhRs. (**A,B**) rSLURP-2 complexes with ‘closed’ binding sites in the α3β2- and α7-nAСhRs. (**C,D**) Two solutions of the rSLURP-2 complex with an ‘open’ binding site in α7-nAСhR. Top and side views of the obtained models are shown. The rSLURP-2 molecule is shown in blue, and its disulfide bonds are indicated in orange. The three loops of rSLURP-2 are labeled. For the α3β2 receptor, the α3 subunits are shown in light green, and the β2 subunits are indicated in wheat. For the ‘closed’ binding site of the homopentameric α7 receptor, the principal and complementary subunits are shown in light green and wheat, respectively. For the ‘open’ binding site of the homopentameric α7 receptor, the principal and complementary subunits are indicated in magenta and light green, respectively. Loops C of the α3 and α7 subunits are indicated in red.

Next, we performed three series of rSLURP-2/nAChR docking: for the α3β2 receptor with a ‘closed’ binding pocket, for the α7 receptor with a ‘closed’ binding pocket, and for the α7 receptor with an ‘open’ binding pocket. Solutions of each docking run were ‘filtered’ by a post-scoring procedure to select the best probable complexes (for details, see Methods). After filtering, 2, 5 and 20 solutions were selected for each series, respectively. The ‘final’ docking solution of each complex was chosen by visual inspection. Representatives are shown in Fig. 7.

In the complex with α3β2 nAChR, rSLURP-2 interacts with the ‘vestibule’ of the ‘classical’ binding site via its loops I and II, with the tip of loop II positioned under loop C of the α3 subunit (Fig. 7A, see Table for a complete list of interactions). In the case of the α7 nAChR with a ‘closed’ binding pocket, rSLURP-2 interacts with the receptor via loops II and III (Fig. 7B), and its extended loop III protrudes into the ‘classical’ binding site. For the rSLURP-2 complex with the ‘open’ binding pocket of the α7 nAChR, two groups of solutions with different ligand orientations were obtained (Fig. 7C,D). In these groups, rSLURP-2 also occupies the ‘vestibule’ of the orthosteric binding site and interacts with the inner side of loop C via loops I and II or loops II and III, respectively.

## Discussion

To investigate the pharmacology of SLURP-2 and to compare it with other human Ly-6/uPAR proteins (SLURP-1 and Lynx1), we studied its activity in several model systems. Affinity purification from cortical brain extracts revealed that rSLURP-2 could bind to a variety of nAChR subunits, including α3, α4, α5, α7, β2, β4, and possibly α6 (Fig. 2), and nAChRs containing these subunits probably represent SLURP-2 targets. A similar pharmacological spectrum has been described previously for ws-Lynx1, which also interacts with multiple α and β nAChR subunits^34^, whereas rSLURP-1 exhibits a selective interaction with the α7 subunit^24^.

Electrophysiological recordings using *Xenopus* oocytes confirmed the ability of rSLURP-2 to interact with α4β2, α3β2, and α7 nAChRs. Similar to ws-Lynx1^29^, rSLURP-2 was observed to inhibit α4β2 and α3β2 nAChRs at micromolar concentrations and to demonstrate concentration-dependent activity at α7 nAChRs (Fig. 3). Enhancement of the ACh-evoked current observed on the dose-response curve for rSLURP-2 (Fig. 3F, dashed circles) resembles the previously reported effects of the competitive inhibitor tubocurarine at α3β4 receptors^35^. The binding of a low concentration of tubocurarine at one site of the receptor has been shown to facilitate ACh-induced opening of the channel. Similar observations and conclusions were obtained in investigations of several cholinergic drugs (atropine, scopolamine, and physostigmine), which demonstrated competitive activity at α4β2 receptors^36^. Enhancement of the ACh-evoked current at low ligand concentrations has also been described for partial agonists of the α7 nAChRs: RG3487^37^ and encenicline EVP-6124^38^.

The current hypothesis for the enhancement (priming) effect of rSLURP-2 at α7 nAChRs is shown in Fig. 3H. These receptors are composed of five identical subunits and are activated by interactions with at least two ACh molecules. In contrast, α7 nAChRs binding by SLURP-2 does not elicit a current through the channel. If the SLURP-2 concentration is low, then only a few or no receptors are occupied by one or two SLURP-2 molecules, and they remain closed. The addition of ACh in the presence of such a low concentration of rSLURP-2 leads to the situation where some fraction of the receptors is occupied by one molecule of ACh and one molecule of SLURP-2. These double-liganded receptors should be activated (designated by the orange core, Fig. 3H), and, on average, more current will flow through the membrane compared with the control. In this case, there are two types of activated receptors: ACh only, and primed by ACh and rSLURP-2. Thus, at low concentrations, rSLURP-2 primes the response to ACh. The inhibition of α7 nAChRs observed at high concentrations of SLURP-2 can be explained either by competition between ACh and SLURP-2 for binding to the receptor or by desensitization of α7 nAChRs upon sustained exposure to rSLURP-2.

The nicotine-induced ERK1/2 MAP kinase phosphorylation assay in PC12 cells revealed that rSLURP-2, but not rSLURP-1 (Fig. 4), could influence nAChR-mediated intracellular signaling via interactions with another possible target– α3β4 nAChR. A similar inhibition of nicotine-induced MAP kinase phosphorylation was recently described for ws-Lynx1^34^, and the results agree well with the pharmacological spectra of these Ly-6/uPAR proteins determined by affinity purification (see above).

Consistent with previously published data^25^, rSLURP-2 slightly promoted the growth of oral keratinocytes (Fig. 5A). Using specific inhibitors of nAChRs, we studied the roles of different nAChRs subtypes in the regulation of epithelial cell proliferation by SLURP-2. The observed increase in the proliferative activity of rSLURP-2 upon α7 nAChR blocking by α-Bgtx indicated that the interaction of SLURP-2 with α7 nAChRs could inhibit cell proliferation. In contrast, the experiments with α-conotoxin MII revealed that the interaction of SLURP-2 with α3β2 nAChRs promoted cell proliferation (Fig. 5B). The data obtained in the presence of non-specific nAChR and mAChR inhibitors (Mec and atropine, respectively) suggested that rSLURP-2 could also interact with muscarinic receptors, but the resulting effect on the keratinocyte proliferation could not be unambiguously characterized on the background of large nAChR-mediated effects. Altogether, the data obtained for keratinocytes suggest a possible role of SLURP-2 at different stages of cell maturation via interactions with various types of acetylcholine receptors. In addition to SLURP-2, keratinocytes also express SLURP-1 and Lynx1 (detected at least at the mRNA level^21^). It was interesting to compare the effects of these Ly-6/uPAR proteins on cell proliferation. While rSLURP-2 promoted the proliferation of Het-1A cells, rSLURP-1 inhibited it^24^ and ws-Lynx1 had no effect (Fig. 5A). Thus, changes in the relative expression level of SLURP-1 and SLURP-2 by keratinocytes may regulate their development.

The ability of rSLURP-2 to interact with M1 and M3 mAChRs was confirmed in competition experiments with the orthosteric agonist NMS (Fig. 6). The observed influence of rSLURP-2 on the rates of NMS dissociation at M1 and M3 mAChRs (Fig. 6D) indicated that rSLURP-2 is an allosteric ligand of the muscarinic receptors. Because oral keratinocytes do not express M1 mAChRs^26^, rSLURP-2 appears to target M3 receptors in these cells. Notably, other Ly-6/uPAR proteins interact with both nAChRs and mAChRs. For example, weak allosteric interactions with mAChRs have been described for ws-Lynx1^29^ and the snake toxin WTX^7^.

NMR data revealed the structural similarity of rSLURP-2 with other Ly-6/uPAR proteins (Fig. 1E). The rSLURP-2 molecule contains a conserved β-structural core with three protruding loops. The loops of rSLURP-2 demonstrate significant conformational plasticity (Fig. 1C,D). The high mobility of the loops has been proposed to be one of the factors underlying the ability of ws-Lynx1 and WTX to interact both with nAChRs and mAChRs, although with rather low affinity (μM range)^7^,^29^. In comparison, snake α-neurotoxins, which have more ordered loops, inhibit nAChRs with high specificity at nM concentrations^39^. The loops of ‘three-finger’ proteins are considered major structural determinants of the interaction with their targets^4^ (nAChRs^40^,^41^, mAChRs^7^,^42^, and GABA_A_ receptors^3^, among others). The sequence comparison of SLURP-2 with other three-finger proteins acting on nAChRs revealed a significantly different charge distribution in the loop regions (Fig. 1A). In contrast to Lynx1 and α-neurotoxins, which are positively charged, the SLURP-2 molecule has an overall negative charge. The altered structural and dynamic properties of SLURP-2 loops in comparison with Lynx1, SLURP-1 and snake α-neurotoxins could imply a different mode of interaction with nAChRs.

Determination of the spatial structure of rSLURP-2 allowed us to model complexes of rSLURP-2 with the extracellular ligand-binding domains of α7 and α3β2 nAChRs (Fig. 7). MD simulation revealed two possible conformations of loop C in the α7 domain providing ‘open’ and ‘closed’ ligand-binding pocket, and only one ‘closed’ conformation of the α3β2 ligand-binding site. Despite the different conformations of loop C and the different orientations of the rSLURP-2 molecule, in all of the modeled complexes, rSLURP-2 interacted via its loops with the inner side of loop C (Table, Fig. 7). Notably, rSLURP-2 loop II participated in complex formation in all cases. The ‘closed’ position of loop C in the α3β2 and α7 domains was found to resemble the receptor conformation in the modeled complex of ws-Lynx1 with acetylcholine-binding protein (AChBP)^29^, a structural homolog of the ligand-binding domain of nAChR, and the X-ray structure of the α7/AChBP chimera complex with the agonist epibatidine^43^. However, in the aforementioned model, ws-Lynx1 interacted with the outer side of the AChBP loop C^29^. The ‘open’ conformation of loop C observed herein for the α7 nAChR domain resembled the position of loop C in the crystal structures of the AChBP and α7/AChBP chimera complexes with antagonists^33^,^44^.

In all of the modeled complexes, the interfaces of the interaction of the α3β2 and α7 nAChRs with rSLURP-2 partially overlapped with the antagonist binding pocket in the structure of the α7/AChBP chimera complex with α-Bgtx^33^ (Table). Thus, the computer modeling results confirmed the possible SLURP-2 targeting to the orthosteric binding site of nAChRs. Comparison of the complexes of rSLURP-2 with α3β2 and α7 nAChRs in the ‘closed’ conformation revealed that the orthosteric binding site of the receptors was occupied by extended loops II and III of the ligand, respectively (Fig. 7A,B). We could speculate that this difference in the binding mode is somehow associated with the difference in rSLURP-2 activity observed at these receptors (Fig. 3). The opposite effects on keratinocyte proliferation mediated by the interaction of rSLURP-2 with the α3β2 and α7 nAChRs could be the result of both different modes of receptor/ligand interactions and different intracellular signaling cascades that are coupled to these receptor subtypes.

In summary, the present data reveal new facets of the activity of SLURP-2. The main finding is the ability of SLURP-2 to interact with multiple molecular targets. SLURP-2 has opposite effects on epithelial cell growth, depending on the subtype of the receptors involved in the interaction. Surprisingly, SLURP-2 demonstrates similar pharmacological properties to the water-soluble analogue of its membrane-tethered isoform Lynx1, despite a low sequence homology. Similar to ws-Lynx1, rSLURP-2 interacts with a broad range of acetylcholine receptors, including the previously unrecognized targets α7, α3β4, α4β2 nAChRs and M1 and M3 mAChRs. This finding contrasts with the pharmacology of another epithelial Ly6/uPAR protein, – SLURP-1, which is a selective non-competitive antagonist of α7 nAChRs^24^.

## Materials and Methods

Recombinant ws-Lynx1, rSLURP-1, rSLURP-2, and ^13^C^15^N-labeled rSLURP-2 were produced in *E. coli* as described previously^27^,^45^,^46^. The purity and homogeneity of the samples were confirmed by HPLC, MALDI-MS, and SDS-PAGE (Supplementary Fig. S1). Disulfide bond formation was confirmed in the reaction with Ellman’s reagent (Sigma-Aldrich). The correct spatial structure for each sample of produced proteins was confirmed by NMR spectroscopy.

### Affinity purification

Human temporal neocortical tissue was obtained from an anterior temporal lobectomy performed in two patients (females, age 30 and 58 years) with medically intractable temporal lobe epilepsy with a hippocampal onset. Written informed consent was obtained before surgery. The study was approved by the Ethical Committee in the Capital Region of Denmark (H-2-2011-104) and performed in accordance with the Declaration of Helsinki. The tissue was immediately frozen on dry ice and stored at −80 °C until use. Neuropathological examinations of the neocortex revealed no abnormalities.

To avoid precipitation, rSLURP-2 was dissolved to 0.2 mM in PBS, pH 5.0 and coupled to PureProteome™ NHS Flexibind magnetic beads (Millipore, Billerica, MA) at a ratio of 1:2 (vol/vol) according to the manufacturer’s instructions to achieve a final rSLURP-2 concentration 66 μM. Successful coupling was confirmed by subsequent protein determination, revealing a substantial decrease in the protein content of the rSLURP-2 solution. Another batch of beads was processed in parallel, but using PBS devoid of rSLURP-2, as a negative control. Prior to use, the beads were incubated in 0.1% bovine serum albumin in PBS, pH 5.0 for 1 hour at 4 °C.

The tissue was lysed in 1 mL lysis buffer (50 mM Tris, 50 mM NaCl, 5 mM EDTA, 5 mM EGTA, 10 μL/mL protease inhibitor cocktail (Sigma-Aldrich), pH 7.5) using a PT1200C polytron blender (Kinematica, Luzern, Switzerland) for 20 seconds. The lysate was centrifuged for 30 minutes at 160,000 × g at 20–22 °C using an air-driven ultracentrifuge (Airfuge^®^, Copenhagen, Denmark), and the supernatant discarded. The pellet was resuspended in 1 mL lysis buffer containing 2% Triton X-100 by blending for 20 seconds, and then incubated for 2 hours at 4 °C on a rotor (15 rpm). Thereafter, the sample was centrifuged as described above, and the resulting supernatant was used for affinity purification. The total protein content was determined using the Pierce 660 nm Protein Assay (Thermo Scientific), and 1000 μg of protein was incubated with 50 μL of magnetic beads in a total volume of 1500 μL of a lysis buffer for 18–22 hours at 4 °C on a vertical rotor (15 rpm). Subsequently, the beads were washed twice with 1 M NaCl, 8 mM Na_2_HPO_4_, 2 mM NaH_2_PO_4_, 0.5% Triton X-100, pH 7.5, three times in 0.1 M NaCl, 8 mM Na_2_HPO_4_, 2 mM NaH_2_PO_4_, 0.5% Triton X-100, pH 7.5 and immediately processed for Western blotting.

### Western blotting

The total protein content was measured using the Pierce 660 nm Protein Assay. Equal amounts of samples were then diluted in loading buffer (120 mM Tris, 20% (v/v) glycerol, 10% (v/v) mercaptoethanol, 4% (w/v) SDS, 0.05% (w/v) bromophenol blue, pH 6.8), incubated for 5 minutes at 95 °C and subjected to gel electrophoresis using AnykDTM gels (Bio-Rad), and blotted onto polyvinylidene fluoride membranes (Bio-Rad). The membranes were washed with Tris-buffered saline with 0.1% Tween 20 (TBS-T) and blocked in TBS containing 5% (w/v) dry milk powder, which was also used for the antibody incubations. Incubation with primary antisera directed against β2 (1:1,000, provided by Dr. Cecilia Gotti), α3, α4, α5, α6, 5-HT_3_ (1:100 #sc-1771, sc-5591, sc-28795, sc-27292, sc-28958 Santa Cruz Biotechnology, Dallas, TX), α7, β4 (1:1,000 #ab23832 and 1:100 #ab156213 Abcam, Cambridge, UK), and pERK1/2 (1:4,000 #9101 Cell Signaling, Leiden, The Netherlands) was performed overnight at 4 °C on parafilm in a humidified container, followed by 3 × 10-minute washes with TBS-T and a 1-hour incubation at 20–22 °C with horseradish peroxidase-conjugated secondary antibody (1:2,000, Dako, Glostrup, Denmark). After thorough washing with TBS-T, enhanced chemiluminescence Western blotting detection reagents (Western Lightning^®^ ECL Pro, Perkin Elmer) were used for signal detection, and the protein bands were visualized using a Chemidoc XR digital image analyzer (Bio-Rad). The membrane was then stripped using Restore Western Blot Stripping Buffer (Thermo Scientific) and reprobed with ERK1 antibody (1:4,000, #610031, BD Transduction Laboratories, Franklin Lakes, NJ). The mean optical densities of the bands were measured, and their corresponding background measurement values were subtracted. Molecular weights were estimated by comparison with dyed protein markers (PageRuler Prestained Protein Ladder, Thermo Scientific).

### Electrophysiology

Two-electrode voltage clamp experiments were performed using *Xenopus laevis* oocytes. The oocytes were prepared and injected as described previously^47^. Briefly, the oocytes were injected with 2 ng of cDNA encoding human α4β2 (ratio 1:1), α3β2 (ratio 1:1), or α7 nAChRs and measured 2–5 days later. All recordings were performed with an automated two-electrode voltage clamp system (HiClamp, MultiChannel System, Germany). The oocytes were clamped at −100 mV and perfused with OR2 (oocyte ringer) containing 82.5 mM NaCl, 2.5 mM KCl, 2.5 mM Ca2Cl, 1 mM MgCl2, 5 mM HEPES, and 20 μg/mL BSA. OR2 was adjusted to pH 7.4. Acetylcholine and rSLURP-2 (stock solution in 100% DMSO) were dissolved in OR2 immediately before use. The data were digitized and analyzed off-line using MATLAB (Mathworks, Natick, MA).

### PC12 cell culturing and ERK1/2 MAP kinase phosphorylation assay

PC12 cells were maintained in 75-cm^2^ flasks coated with 5 μg/mL poly-L-lysine (Sigma-Aldrich) in Dulbecco’s Modified Eagle’s Medium (DMEM, Gibco Life Technologies, NY) supplemented with 10% heat inactivated horse serum, 5% fetal bovine serum, 25 U/mL penicillin, 25 μg/mL streptomycin, 1 mM sodium pyruvate, and 2 mM glutamine at 37 °C in a humidified incubator with 5% CO_2_. The cells were subcultured every 3–4 days by detachment with 0.25% trypsin in EDTA solution (Life Technologies, NY), and re-seeded at 15% confluence.

For the ERK1/2 phosphorylation assay, the cells were seeded in 24-well plates at a density of 12 × 10^4^ cells/cm^2^, 24 hours prior to the experiment. On the day of the experiment, the culture medium was replaced with low-serum medium (1% horse serum, 0.5% fetal bovine serum) for 3 hours, followed by a 10-minute incubation with rSLURP-1 or rSLURP-2 diluted in DMSO/DMEM (final concentration of 1%), followed by a 5-minute stimulation with 25 μΜ nicotine (Sigma-Aldrich). The cells were then lysed in 100 μL of an ice cold lysis buffer/well (100 mM NaCl, 25 mM EDTA, 10 mM Tris, 4 mM Na3VO4, 1 mM NaF, 1% (v/v) Triton X-100, 1% (v/v) NP-40, 1 μL/mL protease inhibitor cocktail, pH 7.4). To ensure complete lysis, the lysates were then placed at −80 °C for 15 minutes, thawed, and sonicated for 5 seconds on ice. The lysates were stored at −80 °C until use. VD-220 peptide (RYHHHDPDGG) and rSLURP-1 at individual concentrations of 10 μM were used as negative controls.

### Experiments with Het-1A cells

Human Het-1 A cells (immortalized line of human oral keratinocytes) were obtained from the American Type Culture Collection (ATCC, CRL-2692). The cells were cultured at 37°C and 5% CO_2_ in BEBM medium (Lonza/Clonetics Corporation, Basel, Switzerland), according to the ATCC recommendations. The culture plates were precoated with a mixture of 0.01 mg/mL fibronectin (Sigma-Aldrich), 0.03 mg/mL bovine collagen type I (Sigma-Aldrich) and 0.01 mg/mL bovine serum albumin (Sigma-Aldrich) dissolved in culture medium. The cells were seeded in 96-well plates (3 × 10^4^ cells per well), and after 24 hours, rSLURP-2 from the 100% DMSO stock solution dissolved in BEBM medium without serum was added to cells. The cells were then incubated for 24 or 48 hours, and the cell viability was characterized using WST-1 reagent (water soluble tetrazolium salt 1, Santa Cruz) or examined under a microscope after staining with Hoechst/propidium iodide.

WST-1 was dissolved in 20 mM HEPES (pH 7.4), and an electron transport reagent, 1-m-PMS (1-methoxy-5-methylphenazinium methyl sulfate, Santa Cruz), was dissolved in deionized water. The solutions were then mixed and added to the plated wells (0.5 mM WST-1 and 20 μM 1-m-PMS per well). Following a 3-hour incubation with WST-1, the cell viability was evaluated spectrophotometrically by measuring the absorbance at 450 nm with normalization to the background at 655 nm (Bio-Rad Spectrophotometer 680, Bio-Rad Laboratories).

The Hoechst/propidium iodide assay allows the evaluation of cell viability by staining the nuclei of all cells with Hoechst 33342 and dead cells with propidium iodide. The cells were stained with 1 μM Hoechst 33342 dye and 0.5 μM propidium iodide. The cell nuclei were visualized using a Nikon Eclipse TS100-f microscope (Nikon Corp) with a 40x lens. The number of cell nuclei was estimated at least in three different wells. Five fields of view (320 × 240 micrometers) were analyzed in each well using the ≪Analyze Particles≫ option available in the ImageJ software. The data obtained by the colorimetric assay with WST-1 (Fig. 5A,B) or by counting the Hoechst-stained nuclei (Fig. 5C and Supplementary Fig. S6) displayed a qualitative correlation with one another. Therefore, the WST-1 data were used for further analysis.

To inhibit nAChRs and mAChRs, the keratinocytes were pre-incubated for 30 minutes with α-Bgtx (Tocris, Bristol, UK), α-conotoxin MII (Tocris, the gift from Dr. S. Khirug and Dr. N. Belevich, Helsinki), mecamylamine hydrochloride (Sigma-Aldrich), or atropine (Sigma-Aldrich), followed by the addition of rSLURP-2.

### Binding of rSLURP-2 to muscarinic acetylcholine receptors

The interactions between rSLURP-2 and mAChRs were analyzed using membranes from CHO cells expressing individual receptor subtypes (kindly supplied by Prof. T.I. Bonner). Preparation and characterization of the cell lines has been described previously^48^. The expression of different mAChR subtypes in these cell lines was confirmed by saturation experiments with ^3^H-NMS (Supplementary Table S2). The expression of the receptor subtypes ranged from approximately 1.5 to 12.1 pmol/mg protein. The non-transfected CHO cells demonstrated only weak, probably unspecific, ^3^H-NMS binding (~0.07 pmol/mg protein). All of the radioligand binding experiments were performed in 96-well plates at 30 °C in medium consisting of 100 mM NaCl, 10 mM MgCl_2_, and 20 mM Na-HEPES, pH 7.4. A final volume of 400 μL was used for the saturation binding experiments or 200 μL for the other experiments. Incubation times of 1 and 2 hours were used for the saturation binding and pseudocompetition experiments, respectively. In the dissociation experiments, the membranes were equilibrated for 2 hours with 3.2 nM ^3^H-NMS with or without 4.2 μM rSLURP-2, and dissociation for the indicated time was then initiated by the addition of 10 μM atropine (final concentration). rSLURP-2 was dissolved in DMSO, which was present during all incubations at a final concentration of 1%. Nonspecific binding was determined in the presence of 10 μM atropine.

The incubations were terminated by fast filtration through GF/B filters (Whatman) using a Brandel harvester (Gaithersburg, MD). The filters were dried, and the retained radioactivity was measured using a solid scintillator Meltilex A (Perkin Elmer) in a Microbeta scintillation counter (Perkin Elmer).

### NMR study

The NMR spectra were acquired on Bruker Avance 600 and Avance 800 spectrometers equipped with cryoprobes. For the resonance assignment and structure calculation, a 0.5 mM sample of ^13^C,^15^N-labeled rSLURP-2 (5% D_2_O, 5% dioxane, pH 5.0, 37 °C) was used. Backbone resonance assignment was determined using the standard set of 3D triple-resonance experiments^49^. 3D ^13^C-HCCH-TOCSY and ^15^N- or ^13^C-filtered 3D TOCSY and NOESY spectra were used for the side chain assignments. The spatial structure calculations were performed in the CYANA program^50^ (Supplementary Table S1). Upper interproton distance constraints were derived from NOESY (τ_m_ 100 ms) cross-peaks via a “1/*r*^6^” calibration. Torsion angle restraints and stereospecific assignments were obtained from ^3^J_H_^N^_H_^α^ and ^3^J_NH_^β^ coupling constants and NOE intensities. Hydrogen bonds were introduced using temperature coefficients of HN protons measured in a temperature range from 20–50 °C (Supplementary Fig. S3). The relaxation parameters of ^15^N nuclei (R1, R2, ^15^N-{^1^H}-NOE, Supplementary Fig. S4) were measured at 60 MHz using the standard set of pseudo 3D experiments. The relaxation parameters were measured for two rSLURP-2 samples (the same sample used for the structure calculation and 0.08 mM of ^13^C,^15^N-labeled rSLURP-2 in water without dioxane). The atomic coordinates for the rSLURP-2 structure have been deposited in PDB under accession code 2N99.

### Computational modeling of rSLURP-2/nAChR complexes

To assess the probable mode of the interaction of rSLURP-2 with its targets, homology models of the extracellular ligand-binding domains of α7 nAChR and α3β2 nAChR were constructed using the crystal structure of the α7/AChBP chimera as a template (PDB Id 3SQ9; Li *et al*.^43^) and MODELLER 8.2 software^51^. The complexes were generated using a customized protein-protein docking procedure subdivided into several steps, similarly to our previous work^7^:

1. 3D models of the ligand-binding domains of α7 and α3β2 nAChRs were subjected to MD simulation in a ≈10 × 10 × 11 nm^3^ water box to produce an ensemble of conformationally distinct states. All simulations were performed using the GROMACS 4.5.2 suite^52^ with established Gromos96 45a3 parameters and the SPC water model. Other MD parameters were as follows: 1 bar of pressure (Berendsen barostat), a temperature of 37 °C (V-rescale thermostat), and PME electrostatics. For each receptor, two independent 200-ns trajectories were calculated and combined.
2. Сonformational clustering was performed using the united 400-ns MD trajectories with the Gromos clustering algorithm and a distance cut-off of 0.25 nm. In the case of the α7 nAchR, different conformations of loop C were observed in the different subunits during the MD simulation. Some of the intersubunit interfaces were in the ‘open’ and some were in the ‘closed’ states. Clustering of the α3β2 and α7 (‘closed’) and the α7 (‘open’) cases yielded 9, 9, and 11 structures, respectively.
3. Analogously, three MD trajectories of 200-ns each were calculated for rSLURP-2 (starting from three different NMR models). Conformational clustering with the same parameters yielded 11 structures.
4. The 9 × 11 = 99, 9 × 11 = 99, and 11 × 11 = 121 protein-protein docking runs were performed for the α3β2/rSLURP-2, α7(‘closed’)/rSLURP-2 and α7(‘open’)/rSLURP-2 systems, respectively. Docking was performed with ZDOCK^53^. At this step, we restricted the interaction of rSLURP-2 to loop C of the receptor. For each run, ZDOCK systematically generated 2000 structures of the complex; the 100 top-scoring structures were used for further analysis (in total: 9900, 9000, and 12100).
5. The selected docking solutions were further ‘filtered’ using an in-house re-scoring protocol. The following criteria were used: (a) rSLUPR-2 has significant contact area with the receptor (>400 Å^2^); (b) the number of “good” contacts (h-bonds, ionic bridges and specific stacking) is greater than 12, and (c) the complementarity of hydrophobic/hydrophilic properties in the complex is >0.6. Analysis of these properties in the complexes was performed with the PLATINUM software^54^ (http://model.nmr.ru/platinum; see ref. 7 for details).

## Additional Information

**How to cite this article**: Lyukmanova, E. N. *et al*. Secreted Isoform of Human Lynx1 (SLURP-2): Spatial Structure and Pharmacology of Interaction with Different Types of Acetylcholine Receptors. *Sci. Rep*. **6**, 30698; doi: 10.1038/srep30698 (2016).

## Supplementary Material

Supplementary Information

## Figures and Tables

**Table 1:** Contacts between rSLURP-2 and α3β2 and α7 nAChRs in the modeled complexes.

rSLURP-2^a^	α3β2, closed	α7, closed	α7, open #1	α7, open #2
Loop I				
THR 7	**(+) CYS 224 (H)** **(+) GLU 225 (H)**			
PHE 9	(−) LYS 188 (H) (−) PHE 144 (T)			(−) TYR 190 (T)
GLY 10				(−) GLY 189 (H) **(+) TYR 115 (H)**
GLY 11	(−) LYS 188 (H)			
Loop II				
ARG 31	(−) ASP 195 (I) (−) ASP 196 (I)		**(+) TYR 217 (H)** (+) GLU 215 (I) (+) ASP 219 (I)	
SER 34		(−) TYR 54 (H) (−) GLN 181 (H)	(+) SER 206 (H)	
ASN 35			(+) ASP 219 (H) (+) THR 221 (H)	(−) ASN 133 (H)
THR 36	(+) ASN 125 (H) **(+) TYR 124 (H)**^b^ **(−) SER 63 (H)**			
GLU 37	**(+) ASN 222 (H)** **(−) SER 63 (H)**			
ASP 38	(−) THR 84 (H)		**(+) ARG 208 (I)**	(+) GLY 175 (H)
LEU 39	**(+) ASN 222 (H)**			**(−) SER 188 (H)** **(+) TYR 115 (H)**
LEU 41				**(−) SER 188 (H)**
LYS 44				(+) GLU 215 (I) (+) ASP 219 (I)
Loop III				
ASP 52		**(+) ARG 208 (I)**^b^	(−) GLN 183 (H) (−) GLU 184 (H)	
ILE 53		**(+) ARG 208 (H)**		
SER 55		**(−) SER 58 (H)** **(−) GLN 79 (H)** **(−) ASP 186 (H)**		
LEU 56		**(+) TYR 115 (H)**		
GLY 57		**(−) GLN 79 (H)**	**(−) SER 188 (H)**	
LEU 58		(−) SER 56 (H) **(−) GLN 79 (H)**	(−) GLY 189 (H)	
GLY 59		**(+) GLU 211 (H)** (−) SER 56 (H)	**(−) SER 58 (H)**	
TYR 61		**(−) GLU 184 (H)**	**(+) TYR 115 (H)** **(+) TRP 171 (H)**^b^ (+) SER 172 (H) (+) TRP 176 (H)	
C-terminus				
ASN 73				**(+) ARG 208 (H)**
ASP 75			(+) LYS 98 (I)	(+) LYS 204 (I) (+) SER 206 (I) (+) **ARG 208 (I)**^b^

^a^(+) denotes the residues of the principal subunit; (−) denotes the residues of the complementary subunit. H, T, and I denote the types of interaction: hydrogen bond, T-shaped stacking, and ionic bond, respectively. Bold residues correspond to the residues of the ligand-binding pocket observed in the X-ray structure of the α-Bgtx complex with chimeric protein AChBP/α7-nAChR^31^.

^b^These contacts were possible upon visualization but were not calculated by the program.
