# Supplementary material for: Secreted Isoform of Human Lynx1 (SLURP-2): Spatial Structure and Pharmacology of Interactions with Different Types of Acetylcholine Receptors
Source: Sci Rep. 2016 Aug 3;6:30698. doi: 10.1038/srep30698 (PMC4971505; doi:10.1038/srep30698)
Supplement: Supplementary Information [file srep30698-s1.pdf]

SUPPLEMENTARY ONLINE DATA

Secreted Isoform of Human Lynx1 (SLURP-2): Spatial Structure and Pharmacology of Interaction with Different Types of Acetylcholine Receptors

E.N. Lyukmanova[1,2,*], M.A. Shulepko[1,2], Z.O. Shenkarev[1,2,3], M.L. Bychkov[1,2], A.S. Paramonov[1,2], A.O. Chugunov[1,2], D.S. Kulbatskii[1,2], M. Arvaniti[4],Eva Dolejsi[5], T. Schaer[6], A.S. Arseniev[1,3], R.G. Efremov[1,7], M.S. Thomsen[4], V. Dolezal[5], D. Bertrand[6], D.A. Dolgikh[1,2], M.P. Kirpichnikov[1,2]

[1]Shemyakin-Ovchinnikov Institute of Bioorganic Chemistry RAS, Miklukho-Maklaya Street 16/10, Moscow 117997, Russian Federation

[2]Lomonosov Moscow State University, Leninskie Gori 1, Moscow 119234, Russian Federation

[3]Moscow Institute of Physics and Technology, Institutskiy Pereulok 9, Dolgoprudny, Moscow Region 141700, Russian Federation

[4]Department of Drug Design and Pharmacology, University of Copenhagen, Jagtvej 160, DK-2100 Copenhagen, Denmark

[5]Institute of Physiology, Academy of Sciences of the Czech Republic (public research institution), Prague, 14220, Czech Republic

[6]HiQScreen Sàrl, 6 rte de Compois, 1222, Vésenaz, Geneva, Switzerland

[7]National Research University Higher School of Economics, Myasnitskaya ul. 20, 101000 Moscow, Russia

*To whom correspondence should be addressed: Ekaterina N. Lyukmanova, e-mail: ekaterina-lyukmanova@yandex.ru, katya@nmr.ru; Phone(fax): +7 495 3306983

SUPPLEMENTARY TEXT

Screening of conditions for NMR study of rSLURP-2.

The protein at concentration above 0.1 mM precipitated at pH > 6.0. At pH values in the range from 3 to 4.5 the two sets of backbone resonances were observed. The one set of signals ($^{1}H^{N}$ dispersion from 7.1 to 9.7 ppm) corresponded to the folded rSLURP-2 and the second one ($^{1}H^{N}$ dispersion from 7.7 to 8.9 ppm) to the unfolded protein. Observed decrease in the dispersion of $^{1}H^{N}$ resonances indicates the unfolding of the β-structural protein core. The population of the folded rSLURP-2 gradually diminished with the decrease of the pH value, and the protein completely turned into the unfolded form at pH ~ 2.5.

At pH ~ 5.0 an increase in the rSLURP-2 concentration above 0.2 mM induced nonspecific protein aggregation. The formation of rSLURP-2 aggregates was accompanied by the protein unfolding reflected in the significant reduction of the $^{1}H^{N}$ signals dispersion. At the protein concentrations between 0.2 and 1.0 mM and pH ~ 5.0 the two structural forms of rSLURP-2 probably corresponding to the monomeric and aggregated protein were simultaneously observed in NMR spectra. At concentration ~ 1.5 mM only the aggregates of unfolded protein were observed.

Simultaneous observation of the NMR signals of the monomeric/folded and aggregated/unfolded rSLURP-2 indicates that the processes of the protein unfolding and aggregation are slow on the NMR timescale and have characteristic times in μs-ms range.

SUPPLEMENTARY TABLES

Table S1. Statistics for the best CYANA structures of SLURP-2 in 5% dioxane, pH 5.0, 37ºC

**Distance and Angle restraints**	
Total NOE contacts	582
intraresidual	185
sequential (|i-j|=1)	213
medium-range (1<|i-j|≤4)	31
long-range (|i-j|>4)	153
Hydrogen bonds restraints (27 bonds, upper/lower)	27/27
S-S bond restraints (5 bonds, upper/lower)	15/15
Torsion angle restraints	81
Angle φ	55
Angle χ1	26
**Total restraints/per residue:**	747/9.8
**Statistics for calculated structures**	
Structures calculated/selected	500/20
Mean CYANA target function (Å^2)	2.25± 0.24
Violations of restraints	
Distance (>0.2 Å)	1
Distance (>0.5 Å)	0
Dihedral angles (>5 °)	1
RMSD (Å)	
Overall	
Backbone	1.78± 0.42
Heavy atoms	2.54± 0.38
Ordered regions (1-7,13-29,42-47,66-72)	
Backbone	0.46 ± 0.08
Heavy atoms	1.21 ± 0.17

Table S2. Parameters of ^{3}H-NMS binding to membranes from non-transfected CHO cells (CHO-K) and CHO cells expressed M1, M2, M3, M4, and M5 subtypes of mAChR determined in saturation experiments.

	**CHO-K**	**CHO-M1**	**CHO-M2**	**CHO-M3**	**CHO-M4**	**CHO-M5**
Kd (pM)		198±13	525±48	187±12	125±9	257±22
Bmax (pmol/mg prot)	0.07±0.01	4.2±0.6	12.1±2.1	3.0±0.7	3.5±0.8	1.5±0.4

Bmax values for CHO-K membranes represent total binding. Specific binding, if any, would be even lower. Results are the mean ± S.E. of four independent membrane preparations (n=4).

SUPPLEMENTARY FIGURES

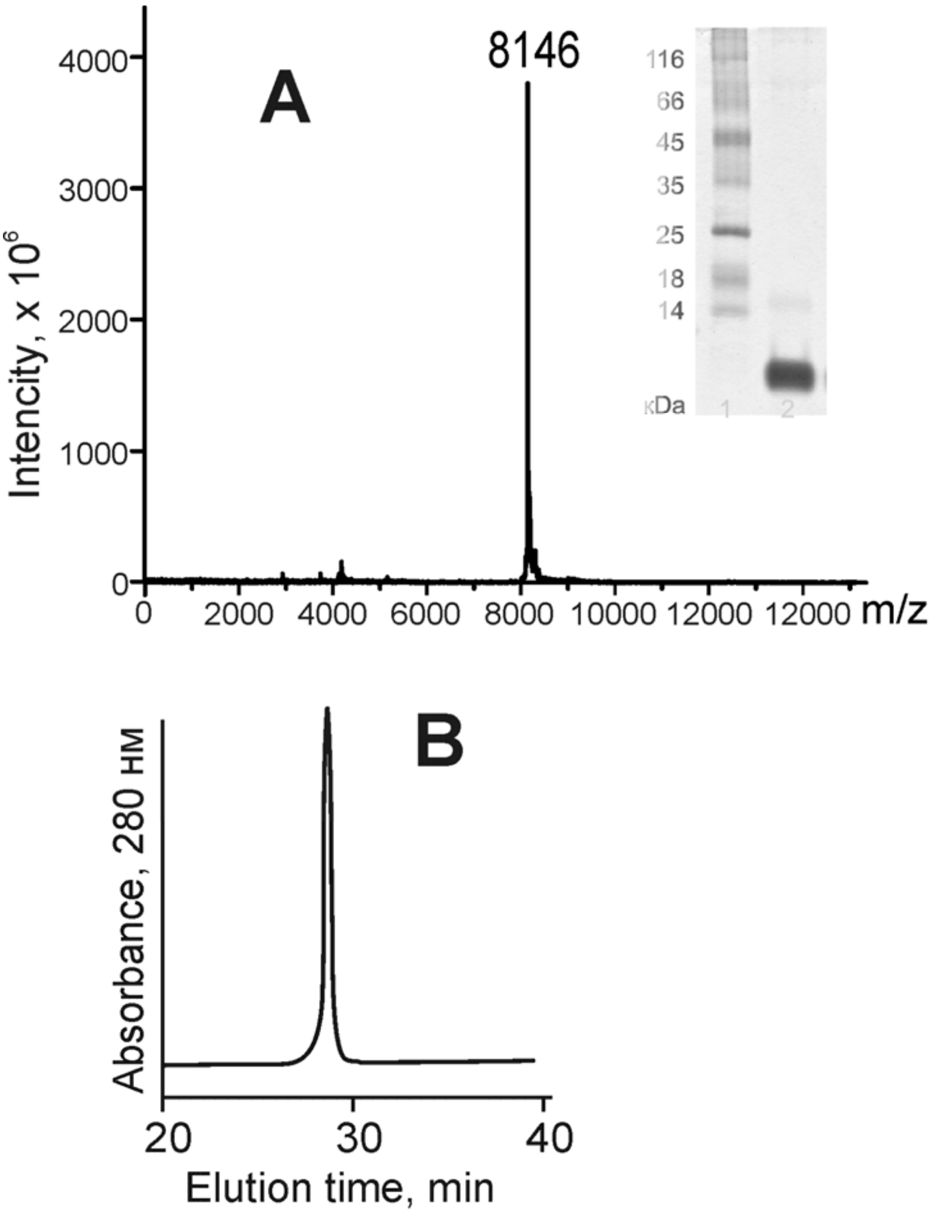

Figure S1. Characterization of recombinant rSLURP-2 by MALDI mass-spectrometry (A), SDS-PAGE (insert), and HPLC (B).

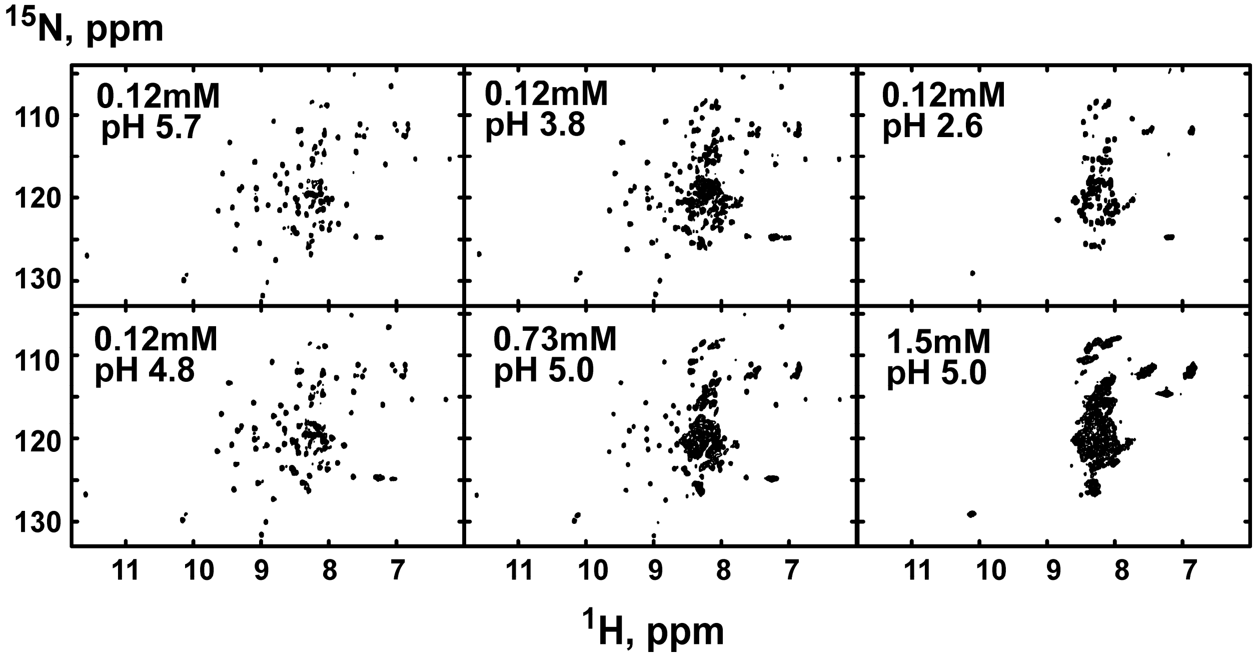

Figure S2. Screening of conditions for NMR study of rSLURP-2. 2D ^{1}H-^{15}N-HSQC spectra of ^{13}C,^{15}N-labelled rSLURP-2 at 37°C and different pH and concentrations are shown.

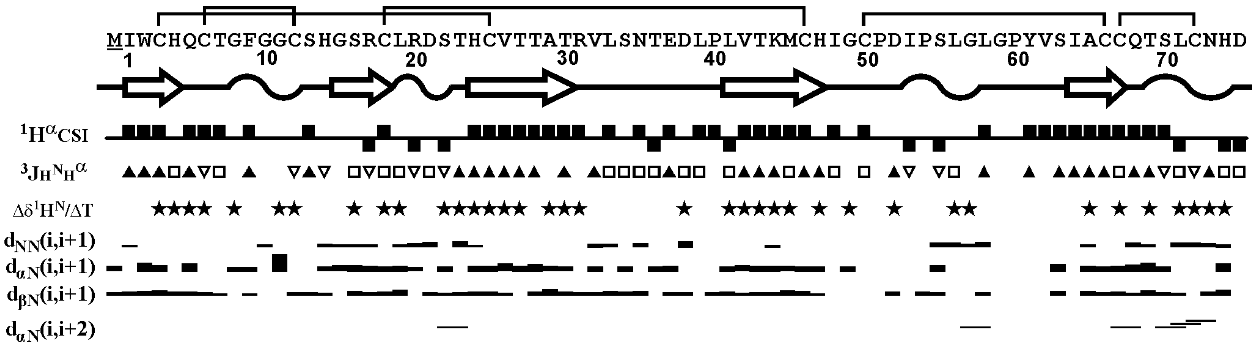

Figure S3. Overview of NMR data collected for 0.5 mM rSLURP-2 (5% dioxane, 5% D_2O, pH 5.0, 37ºC). H^{α} chemical shift indices (CSIs), $^{3}J_{H^{N}H^{\alpha}}$ coupling constants, temperature coefficients of amide protons ($\Delta\delta H^{N}/\Delta T$), NOE connectivities are shown versus the peptide sequence. The positive and negative values of CSIs denote a β-strand and α-helical propensity, respectively. Large (> 8.5 Hz), small (< 5 Hz) and medium (others) $^{3}J_{H^{N}H^{\alpha}}$ couplings are designated by the filled triangles, open triangles and open squares, respectively. $\Delta\delta^{1}H^{N}/\Delta T$ values were measured in a temperature range 20 - 50ºC using 2D ^{15}N-HSQC spectra. The stars denote amide protons with temperature gradients less than -4.5 ppb/K. The NOE connectivities are denoted as usual. The widths of the bars correspond to the relative intensity of the cross-peak in the 100 ms NOESY spectrum. Elements of secondary structure are shown on a separate line; the β-strands are designated by arrows and tight β/γ-turns by wavy lines.

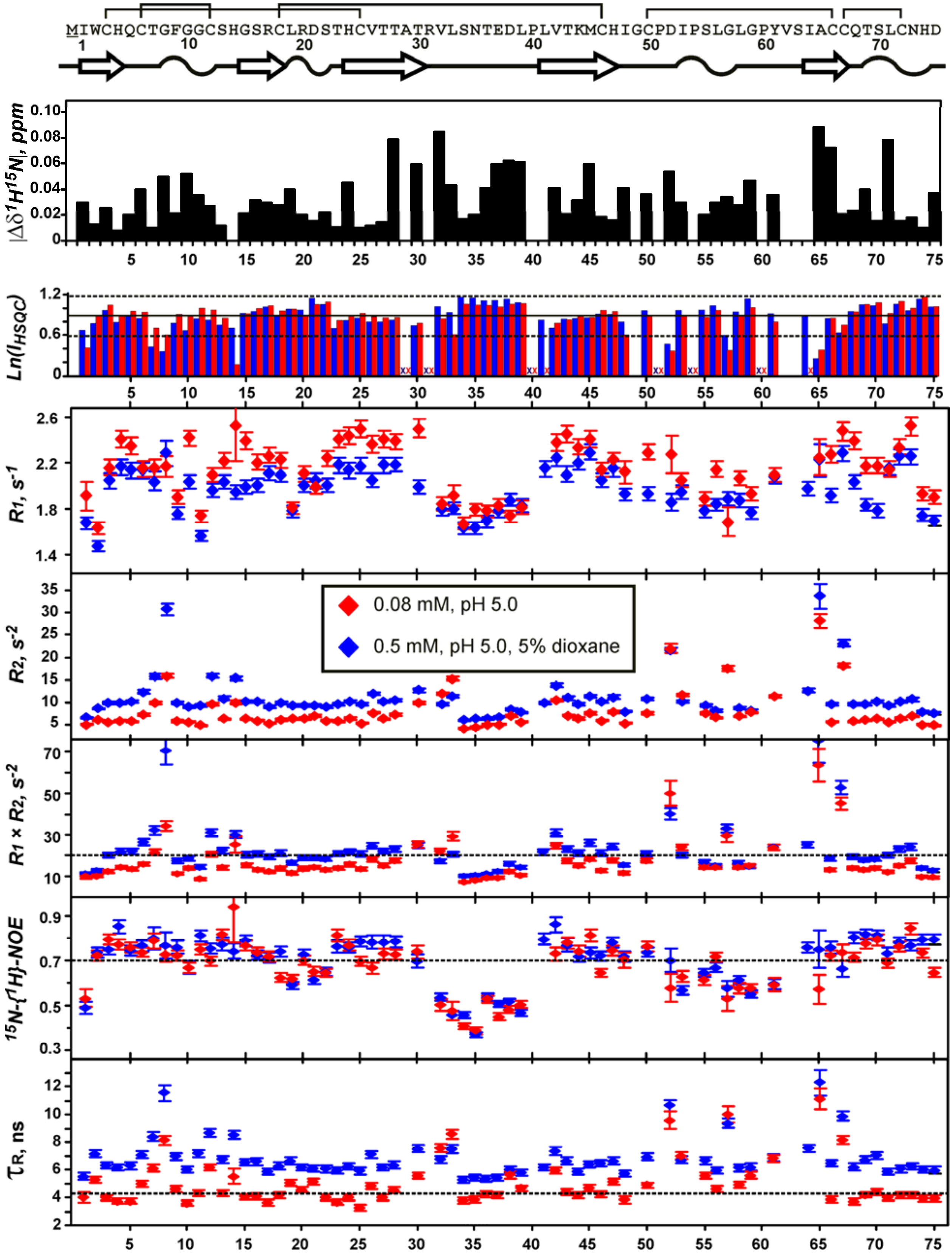

Figure S4. Signal intensities in the HSQC spectra (Ln[I_{HSQC}], logarithmic scale) and ^{15}N relaxation data measured at 60 MHz are shown for two rSLURP-2 samples: (Blue) 0.5 mM, 5% dioxane, pH 5.0, 37ºC; and (Red) 0.08 mM, pH 5.0, 37ºC (without dioxane addition). The

normalized difference of $^1H^N$ and $^{15}N^H$ chemical shifts $\left(\sqrt{(\Delta\delta^1H^N)^2 + (\Delta\delta^{15}N^H/5)^2}\right)$ between the rSLURP-2 signals in the two samples is shown on the top. On the HSQC intensity plot the crosses denote overlapped peaks and Pro residues. The value corresponding to average intensity (calculated for the 0.08 mM sample) is shown by solid line and twice larger and lower values are shown by dashed lines. Residues displaying NOE < 0.7 for the 0.08 mM sample are subjected to extensive motions in ps-ns timescale. Residues displaying $R_1 \cdot R_2 > 20$ s^{-2} [Kneller, J.M., et al. *J.Am.Chem.Soc.* **124,** 1852-1853 (2002)] or demonstrating the low intensity of the HSQC cross-peaks (including the signals broadened beyond the detection limit) for the 0.08 mM sample are subjected to exchange fluctuations in μs-ms timescale (including fluctuations induced by dynamic formation and disruption of the protein aggregates). The effective rotational correlation time (τ_R) values were calculated from the R_2/R_1 ratios. The level corresponding to average value (4.2 ns) calculated over "stable" regions (NOE > 0.7, $R_1 \cdot R_2 < 20$ s^{-2}) of the protein for the 0.08 mM sample is shown by dotted line. The average τ_R value for 0.08 mM rSLURP-2 is nicely corresponds to the τ_R value (4.1 ns) calculated for the monomeric WTX[P33A] from ^{15}N relaxation data (70 MHz, pH 3.0, 30 C) [Lykmanova, E.N., et al. *J.Biol.Chem.* **290,** 23616-23630 (2015)]. This indicates that rSLURP-2 is in monomeric state in aqueous solution at concentration of 0.08 mM.

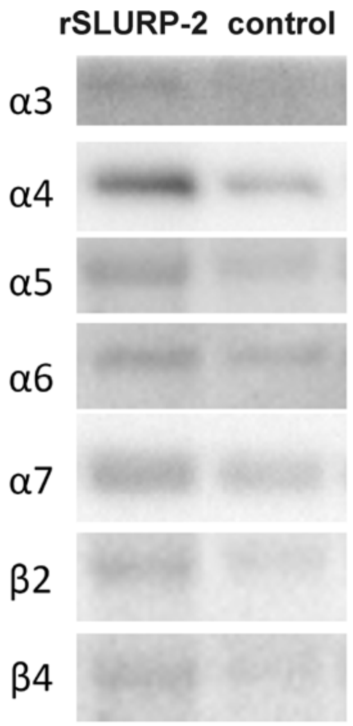

Figure S5. Affinity purification of different nAChR subunits from human temporal cortical homogenates with rSLURP-2 that was covalently coupled to magnetic beads or non-coupled beads (control). The representative blot images from the second independent experiment are shown.

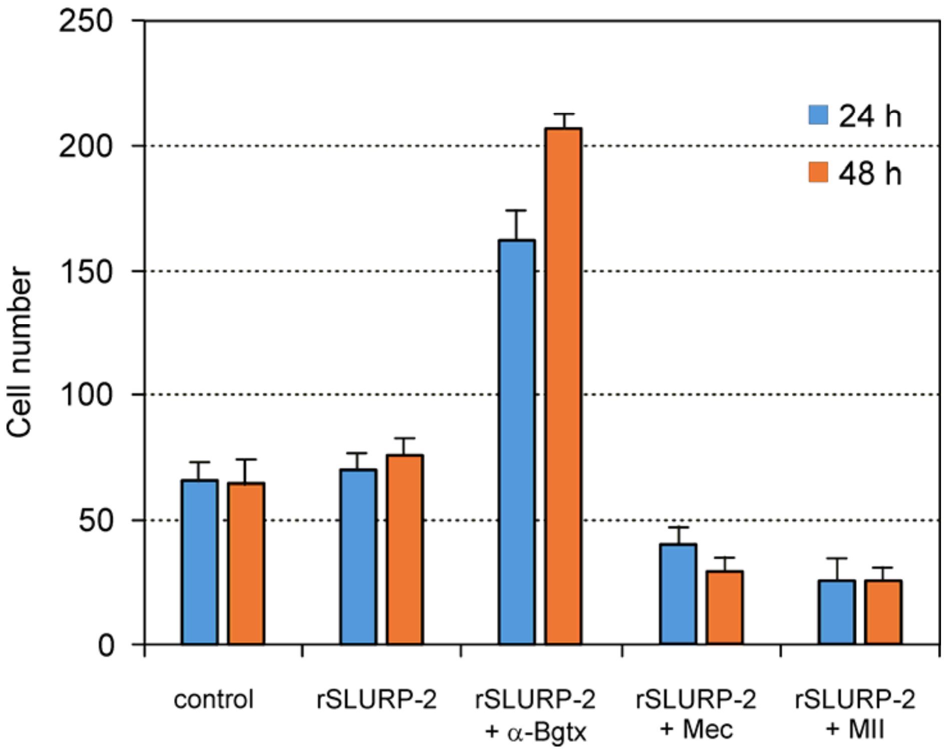

Figure S6. The number of Het-1A cell nuclei after 24- and 48-hour incubation with rSLURP-2 and different nAChR inhibitors. Control is the experiment without rSLURP-2 and inhibitors. The cell nuclei were visualized after staining with 1 μM Hoechst 33342 using a Nikon Eclipse TS100-f microscope (Nikon Corp) with a 40x lens. The number of cell nuclei was estimated at least in three different wells from 96-well plates. Five fields of view (320×240 micrometers) were analyzed in each well using the «Analyze Particles» option available in the ImageJ software. Data points are means ± S.E. of the values obtained in three independent experiments.
